# Aggregation behavior of newly synthesized Gemini cationic surfactants in absence and in presence of different inorganic salts in 15% DMSO–water solvent

**DOI:** 10.1038/s41598-024-69559-1

**Published:** 2024-09-02

**Authors:** Farid I. El-Dossoki, Mohamed A. Migahed, Mahmoud M. Gouda, Samir Abd El Hady Abd El-Maksoud

**Affiliations:** 1 Chemistry Department, Faculty of Science, Port-Said University, Port-Said, Egypt; 2 Department of Petroleum Applications, Egyptian Petroleum Research Institute (EPRI), Cairo, Egypt

**Keywords:** Chemistry, Chemistry publishing, Physical chemistry, Surface chemistry, Chemical synthesis

## Abstract

In this study, three Gemini cationic surfactants related to thiazol-2-amine with three hydrocarbon chain lengths including 3-hexylthiazol-3-ium (TAC6), 3-dodecylthiazol-3-ium (TAC12) and octadecylthiazol3-ium (TAC18) were prepared. Surfactant structures were confirmed with IR and ^1^HNMR Spectroscopies. Critical micelle concentrations for all surfactants in 15% DMSO-Water solvent were measured using conductometric, refractometric, and densitometric techniques. Thermodynamics parameters were computed and explained. Also, enhancing properties of all surfactants were indicated under the effect of two concentrations, 0.001 M and 0.01 M, of six inorganic salts including Cl^−^, Br^−^, I^−^, Co^+2^, Cu^+2^, and Mn^+2^ radicals using conductivity and refractive index measurements. All techniques used to measure critical micelles concentration showed a good convergence in measuring CMC values and the behavior of all surfactants in 15% DMSO-water solvent. Increasing the binding constant of the counter ion and association constant reflects the effect of hydrocarbon chain length increment on enhancing micelle formation, where TAC 18 was shown as the lowest CMC in all applied measurements. Modeling the density of all surfactant solutions under study indicates an increase in hydrophobic polarizability with an increase in the molecular weight of the surfactant. Inorganic salts decreased the CMC of all surfactants with the increase in Gibbs free energy of micellization which ensures easier formation of more stable micelles in the presence of a salt solution. The effect of salts on decreasing CMC for all surfactants under study was arranged in the following order: Mn^+2^ < Cu^+2^ < Co^+2^ for cationic radicals and I^−^ < Br^−^ < Cl^−^ for anionic radicals.

## Introduction

Gemini cationic surfactants are essential in many industrial domains^1^, including the transfer of genetic materials, oil recovery, food processing, antimicrobials, cosmetics, and corrosion inhibitors^2–7^. Turning surfactants into Gemini type enhances their benefits by lowering their critical micelle concentration (CMC)^8^, increasing their solubility in water, producing unique micelle structures and aggregation behavior, and improving their efficacy in lowering the interfacial tension^9^. The particular structure of Gemini cationic surfactant is comprised of two hydrophilic heads and two hydrophobic tails that are connected by spacers with different chemical compositions, situated at or near the head group levels^10^. The chemical structure of Gemini cationic surfactant gained the primary effect influencing surfactant adsorption at the air/water interface and aggregation in aqueous solution. Physicochemical and biological properties can be precisely tailored to suit a variety of applications by altering structural moieties including spacers, head groups, and hydrocarbon chains^9,11^.

Several techniques were used to measure the CMC of Gemini cationic surfactants including conductivity, surface tension, density, refractive index, viscosity, and spectrophotometric^12–20^. Thermodynamic properties including; molar volume (V_ϕ_), polarizability (α_D_), and ionic association constant (K_a_) were used to understand the behavior of solvation and micellization mechanism of surfactants^21–23^. Aggregation properties of surfactants affected by different factors include the nature of surfactants (hydrophobic chain length, and hydrophilic head group area), temperature, solvent, additive, pressure, pH, and ionic strength of surfactant solutions^24–28^.

The current article aims to synthesize and characterization of three Gemini cationic surfactants related to thizol-3-ium bromide. Accurate determination of their CMC values uses several methods, including conductivity, refractive index, density, and molar volume. Also, the related thermodynamic parameters were indicated and explained. The parameters include; binding constant of counter ions (β), dissociation constant (α), Gibbs free energy of micellization (ΔG_mic_) and association (ΔG_ass_), molar refraction (R_m_), polarizability (α_D_), electrostriction volume (V_E_) and Vander Waals volume (V_w_). Also, simulating the effect of two concentrations of six different inorganic salts on the properties of these Gemini cationic surfactants. The current work also attempts to investigate changes in the thermodynamics of solvation and micellization of synthesized surfactants into a 15% DMSO-Water solvent under the effect of different salts.

## Experimental

### Materials

All chemical compounds used in this study were purchased from Sigma-Aldrich, an international company based in the United States, with a high purity grade, eliminating the need for additional purification.; terephthalaldehyde (99%), thiazol-2-amine (99%), bromohexane (98%), bromododecane (97%), bromooctadecane (97%), acetone (99.9%), ethanol (99.9%), dimethyl sulfoxide (DMSO) (99.9%), sodium chloride (98%), sodium bromide (98%), sodium iodide (98%), cobalt chloride (98%), manganese chloride (98%), copper chloride (98%) and bi-distilled water from our lab (conductivity, κ_s_ < 2 µS cm^−1^).

### Synthesis of gemini cationic surfactants

**Scheme 1:**
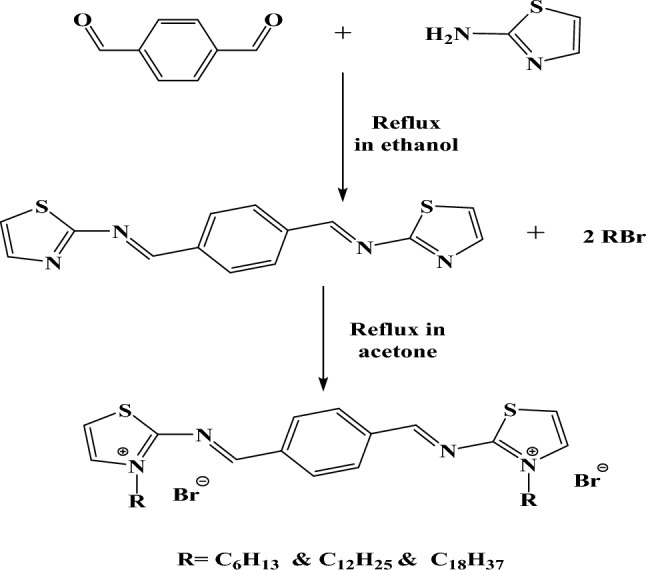
Synthetic route of TAC Gemini cationic surfactants.

Terephthalaldehyde (0.1 mol) was refluxed with thazol-2-amine (0.1 mol) for 12 h in the presence of ethanol (100 mL) as a solvent and 0.01% (by weight) of p-toluene sulfonic acid as a dehydrating agent. The reaction mixture was left to cool overnight and then filtered. The products were recrystallized twice from ethanol, washed with water, and dried under vacuum at 60 °C to afford crystals of (1E,1′E)-1,1′-(1,4-phenylene)bis(N-(thiazol-2-yl)methanimine). The product was refluxed with (0.2 mol) of different alky halides (R = C_6_H_13_ & C_12_H_25_ & C_18_H_37_) in acetone for 24 h. The reaction mixture was left to cool overnight and then filtered. The products were recrystallized twice from acetone, washed by water and dried under vacuum at 60 °C to afford crystals of 2,2′-(((1E,1′E)-1,4-phenylenebis(methaneylylidene))bis (azaneylylidene))bis(3-hexylthiazol-3-ium) bromide (TAC 6); 2,2'-(((1E,1'E)-1,4-phenylenebis(methaneylylidene))bis(azaneylylidene))bis(3-dodecyl thiazol-3-ium) bromide (TAC 12); and 2,2′-(((1E,1′E)-1,4-phenylenebis(methaneylylidene)) bis(azaneylylidene))bis(3-octadecylthiazol-3-ium) bromide (TAC 18) as shown in (Scheme 1).

### Characterization of the Gemini cationic surfactants

The Thermo-Fisher Nicolet iS10 IR spectroscope (United States) was utilized to characterize the synthesized surfactants (TAC 6), (TAC 12), and (TAC 18) within the range 400–4000 cm^-1^, with a resolution of 4 cm^−1^ where potassium bromide pellets were combined with all solid surfactants. Based on the synthetic surfactant Structure, certain functional groups were demonstrated by the wavelengths of the peaks. Next, proton nuclear magnetic resonance spectroscopy (^1^H-NMR), United States, was used to characterize all surfactants. DMSO was used as a solvent during sample preparation, and a 500 MHz JNM-ECA Series FT-NMR spectrometer was used to capture the results. The structures of the samples were used to express the chemical changes in parts per million.

### Solvation studies of the synthesized Gemini cationic surfactants

4510 Jenway conductivity meter 0–1.999S (UK) with certainty ± 0.050 µS cm^−1^ and temperature accuracy 0.1 °C had been calibrated with a measuring cell constant (K_cell_ = 1 cm^−1^) by using several standard potassium chloride solutions at 298.15 K^29^, then used to take all conductivity measurements. The concentration of TAC 6, TAC 12, and TAC 18 solutions was prepared with 0.001 M solution in 15% DMSO-water solvent. Surfactant solutions progressively were added to 15% DMSO-water solvent, in the absence and the presence of two different concentrations (0.001 M and 0.01 mol^−1^L) of six inorganic salts. All components were placed in a multi-port glass cell. The recirculation thermostat “ultraterm 200” adjustable temperatures from ambient + 5 to 200°c (JP Selecta, Spain) was utilized to regulate the desired temperature within the cell. The liquid was thoroughly mixed after each addition to ensure uniform mixing, and the conductivity meter with epoxy-bodied laboratory Conductivity Electrode was then used to measure it. Specific conductance measurements were made three times, and the average was used to calculate the observed results. Density measurements were performed using one milliliter from 15% DMSO-water solvent and surfactant solutions in the 15% DMSO-water solvent after each addition. The refractive index has been determined with a portable digital refractometer from Mettler Toledo (Refracto 30GS) (± 0.0001) (United States). The reference samples that came with the refractometer were used to calibrate it^30^. Attention optical tensiometer theta lite, Biolin scientific, China (Measuring range 0.01–2000) (Accuracy ± 0.01 mN/m) with one attention software was used to measure surface tension (γ) through the contact angle of different concentrations of all examined surfactants.

## Results and discussion

### Structure confirmation of Gemini cationic surfactants

The chemical structure of the synthesized Gemini cationic surfactants was indicated using ^1^HNMR spectroscopy and IR spectroscopy.

#### ^1^H-NMR analysis

**Figure 1:**
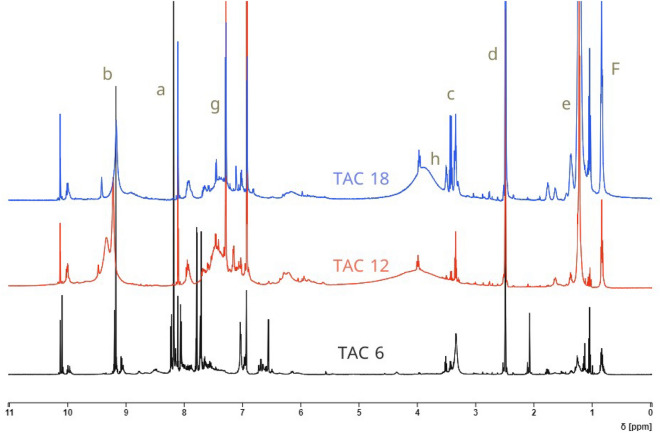
^1^HNMR spectrum of all surfactants in DMSO solvent.

The structure of TAC 6 surfactant, as an example of newly synthesized surfactants, with different position symbols (a−h) was shown in Structure 1. As shown in (Fig. 1), chemical shifts corresponding to different functional groups were indicated including peaks at 0.84–0.85 (s, 1H, (f) –CH_3_); 1.22–1.23 (t, 6H, (e) –CH_2_); 2.49 (d, 4H, (d) –CH_2_); 3.34–3.40 (s, 2H, (c) -CH_2_); 9.20 (s, 1H, (b) –N = CH–R); 8.02–8.10 (s, 2H, (a) –CH); 7.29–7.78 (t, 3H, (g) –CH); 3.43–3.99 (s, 1H, (h) –CH–S.

**Structure 1:**
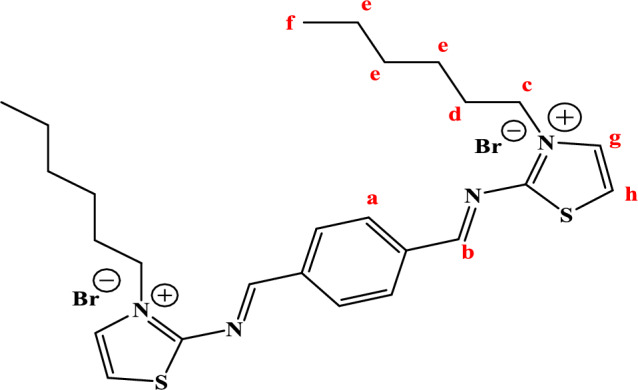
Chemical structure of TAC 6 with numbered at different positions (**a**–**h**)

#### IR analysis

**Figure 2:**
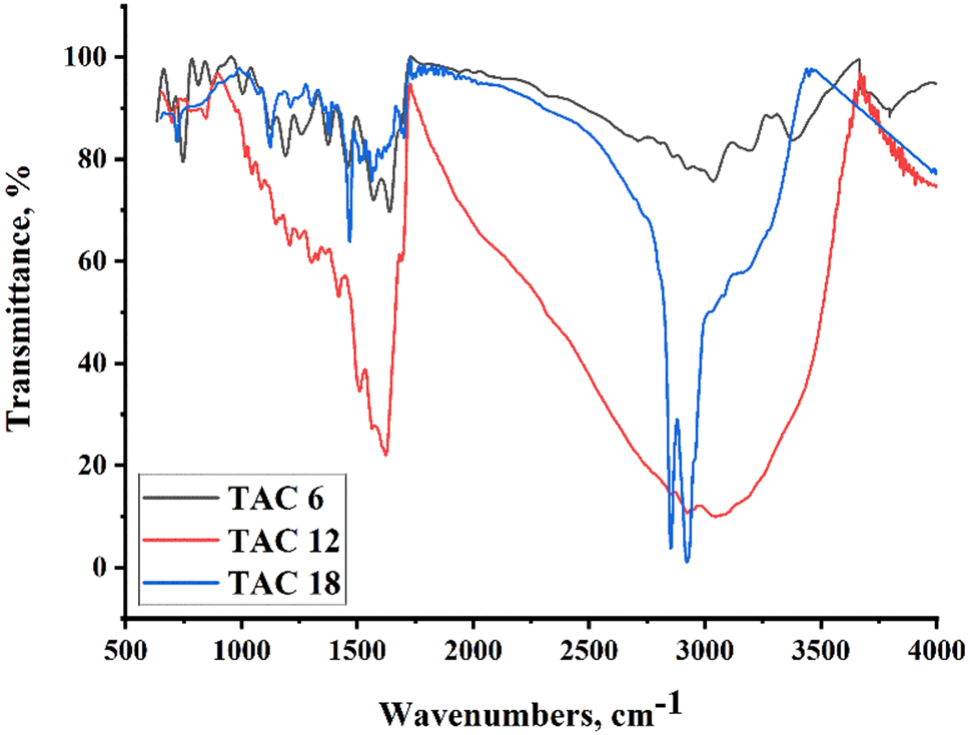
IR spectra for All Gemini cationic surfactants TAC 6, TAC 12 and TAC 18.

The IR spectrum of TAC 6, TAC 12, and TAC 18 Gemini cationic surfactants is represented in (Fig. 2). All vibration bands as functional groups were represented at the following wavelength where; vibration –CH in benzene ring 2843–2889 cm^−1^, –CH in methyl group or aliphatic chain at 2917–2933 cm^−1^, S–CH=C at 3170–3197 cm^−1^, –CH_2_ in the aliphatic chain at 3009–3041 cm^−1^, CH_2_–CH_2_ or N–CH= at 1620–1644 cm^−1^, benzene –CH=N or CH=CH at 1506–1559 cm^−1^, = C–CH between the benzene ring and thiazole ring at 1411–1427 cm^−1^, N^+^–CH = in thiazole ring at 1306–1374 cm^−1^, CH_2_ in the aliphatic chain at 722–742 cm^−1^.

### Solvation studies

#### Critical micelle concentration detection

**Figure 3:**
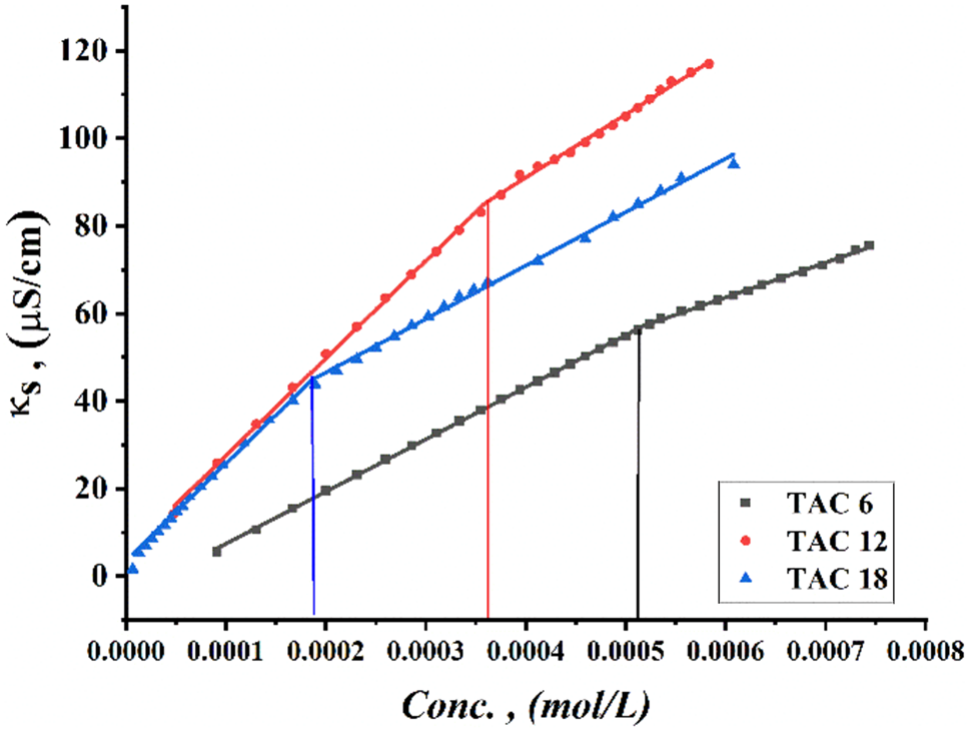
Conductivity vs. molar concentration for all surfactants TAC 6, TAC 12 and TAC 18 in 15% DMSO-water at 298.15 K.

**Figure 4:**
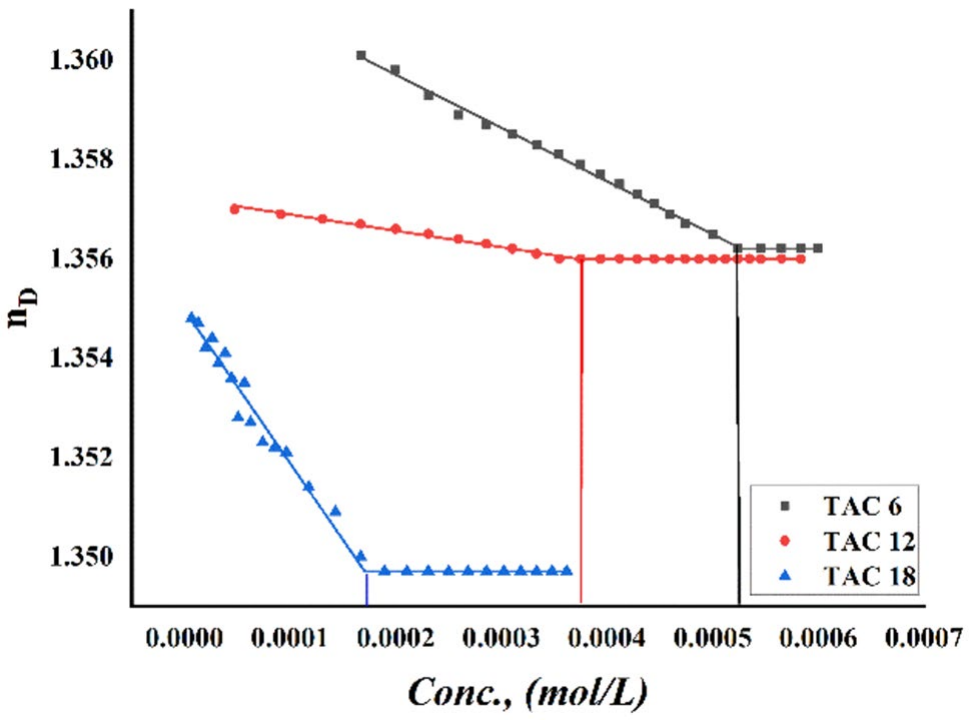
Refractive index vs. molar concentration for all surfactants TAC 6, TAC 12, and TAC 18 in 15% DMSO-water at 298.15 K.

**Figure 5:**
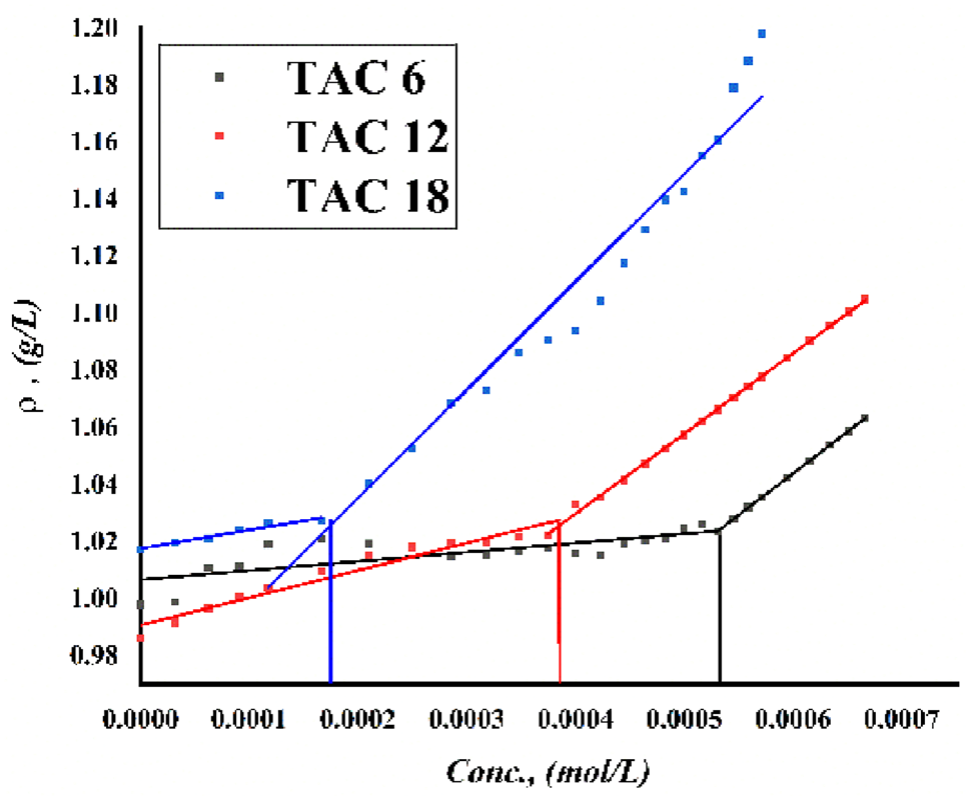
Densities vs. molar concentration for all surfactants TAC 6, TAC 12 and TAC 18 in 15% DMSO-water at 298.15 K.

**Figure 6:**
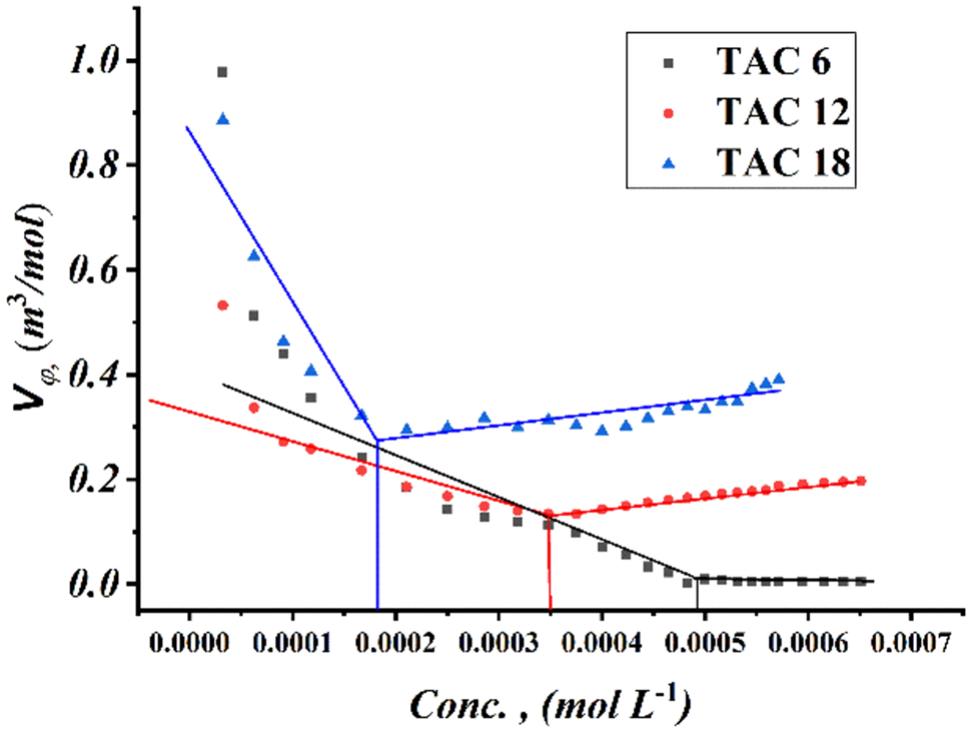
Molar Volume vs. molar concentration for all surfactants TAC 6, TAC 12, and TAC 18 in 15% DMSO-water at 298.15 K.

**Figure 7:**
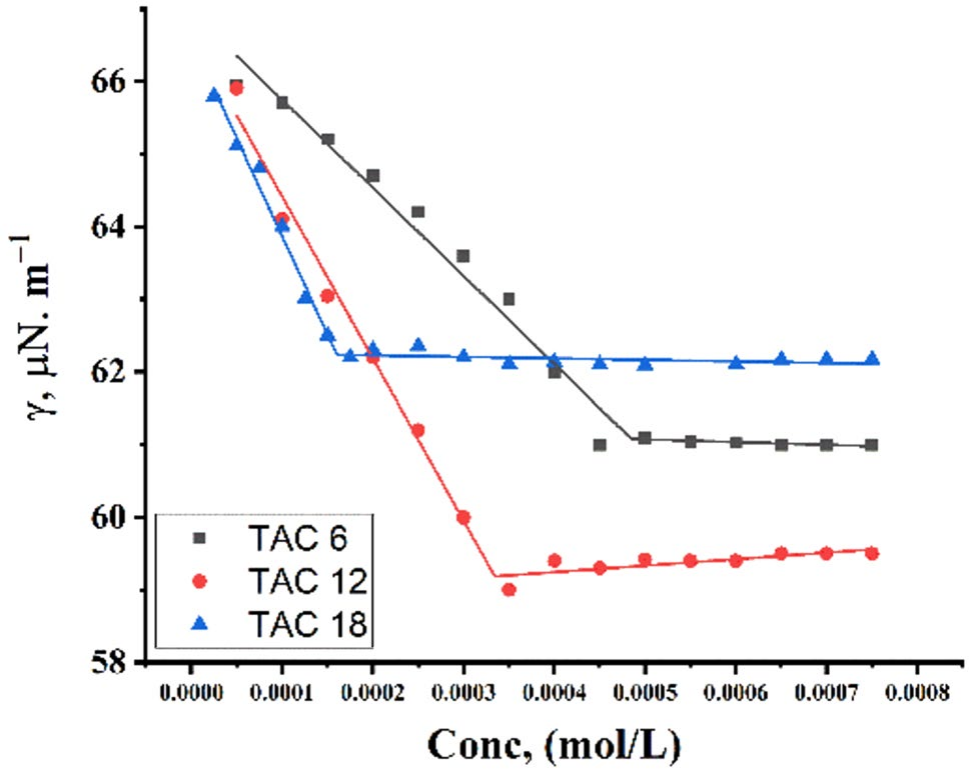
surface tension vs. molar concentration for all surfactants TAC 6, TAC 12, and TAC 18 in 15% DMSO-water at 298.15 K.

Critical micelles concentration values in 15% DMSO-water solvent at 298.15 K for all surfactants under study were measured by several techniques including; conductometric measurement (Fig. 3), refractometric measurement Fig. 4, densitometric measurement (Fig. 5), molar volume (Fig. 6) and surface tension (Fig. 7) where concentration in (mol L^−1^) plotted against different parameters; specific conductance in (µs/cm), refractive index, density (g/cm^3^), molar volume (m^3^/mol) and surface tension (mN/m), of the solution after each addition.

The CMC values for all surfactants under study; TAC 6, TAC 12, and TAC 18 in 15% DMSO-water solvent at 298.15 K estimated from different techniques (conductivity, refractive index, density, and molar volume) were summarized in (Table 1).

**Table 1:** CMC for all surfactants in 15% DMSO-Water solvent at 298.15 K with different techniques.

Surfactant abbreviation	Conductivity (mol L^−1^)	Refractive index (mol L^−1^)	Density (mol L^−1^)	Molar volume (mol L^−1^)	Surface tension mol L^−1^
TAC 6	0.000534	0.000526	0.000530	0.000492	0.000504
TAC 12	0.000363	0.000370	0.000387	0.000350	0.000343
TAC 18	0.000180	0.000171	0.000167	0.000184	0.000166

The formation of micelles in each surfactant solution was indicated from the sharpening decrement in the rate mobility of monomers and dimers surfactants, which was indicated by a slow increase in the specific conductance after the CMC for all surfactants under study; TAC 6, TAC 12 and TAC 18 as shown in (Fig. 3) which indicate the formation of micelles^31,32^.

The refractive indices of all surfactants under study; TAC 6, TAC 12, and TAC 18 shown in (Fig. 4) indicate a decrease with the increment in concentration of each surfactant. This may be related to the solvation of hydrophobic hydrocarbon chains of all surfactants until they reach their CMC value where dehydration occurs^33^.

Density is another physical characteristic of surfactant solutions that varies depending on the surfactant aggregation state. Furthermore, the molecular weight of the surfactant and the various hydrophobic solvation grades influence how much the density increases in the dimer state. In particular, as shown in (Fig. 5), the density of a solution of all surfactants TAC 6, TAC 12, and TAC 18 increased more in the micelle state per unit mass of the surfactant than it did in the dimer state. The decrement in the solvation between all surfactants under study and 15% DMSO-water solvent led to a decrease in the molar volume of the surfactants in the dimer state. The increase in the density through the surfactants in dimer form is due to the increase in molecular weight through surfactants in the following arrangement TAC 18 < TAC 12 < TAC 6 per unit volume^34^. After critical micelles concentration of all surfactant where dimers left bounded water free through dehydration process^12^ which led to approximately constant values of the molar volume of all surfactant solutions as shown in (Fig. 6). The high increment in molecular weight of all synthesized surfactants with constant values of molar volume led to a sharp increment in all surfactant densities after CMC^35,36^.

Surface tension measurement is commonly used to determine the Critical Micelle Concentration (CMC) of surfactants. Gemini cationic surfactants adsorb at the interface between solution and air, forming a monolayer that reduces the cohesive forces among solution molecules^37^. This adsorption increases until the surfactant concentration reaches CMC, after which micelles begin to form in the solution, reducing their presence at the solution surface as shown in (Fig. 7). However, our study found that surface tension measurement was not effective in determining CMC for all surfactants. This limitation may stem from the solvent composition used to dissolve the examined surfactants TAC 6, TAC 12, and TAC 18, which consisted of a 15% DMSO-water mixture^38^. Dimethyl sulfoxide (DMSO), a polar aprotic solvent, is known to potentially inhibit the reduction of surface tension^39^. It can dissolve non-polar Gemini cationic surfactants, thereby disrupting their micellar structure and reducing their surface activity^40^. Moreover, DMSO’s high dielectric constant may compete with Gemini cationic surfactants for interface adsorption, further impacting the surface tension reduction process.

**Figure 8:**
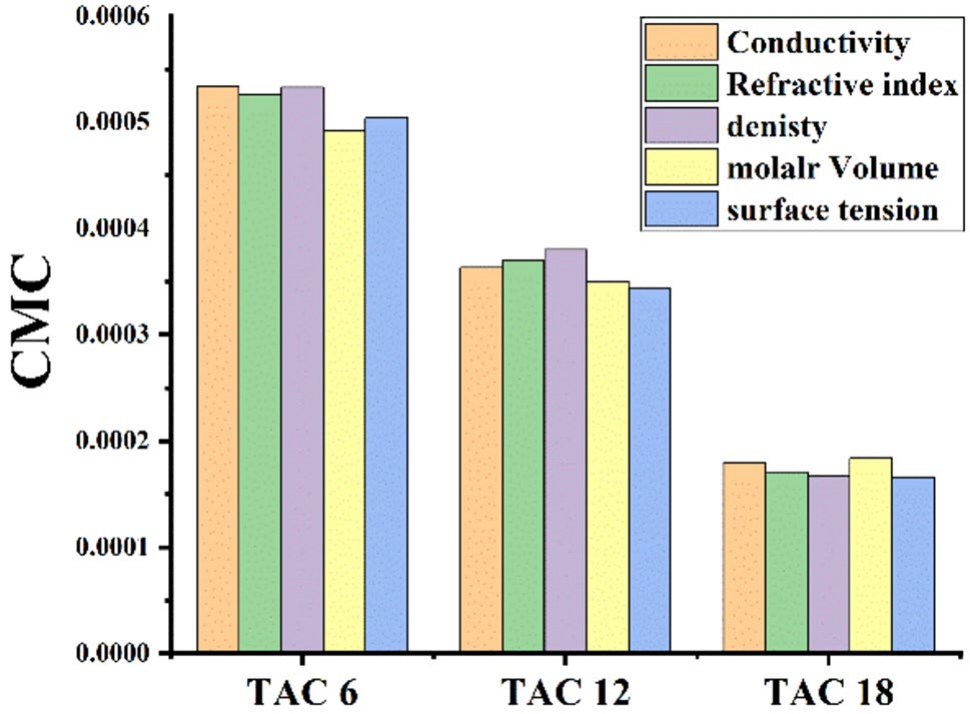
Comparison between CMC measuring from different techniques.

Techniques used for all synthesized surfactants under study; TAC 6, TAC 12, and TAC 18 indicate the change in properties of all surfactants with increments in surfactant concentration. These techniques were used to observe the solvation mechanism of all synthesized surfactants in 15% DMSO-water solvent. Where critical micelles concentration of all surfactants were measured. There was a good fitting between measuring the CMC of all surfactants with different techniques. All techniques showed good agreement in explaining solvation and micellization of all surfactants through concentration as shown in (Fig. 8).

#### Thermodynamic parameters from conductivity measurements

Estimation of the degree of ionization (α) and the counter ion binding (β) for TAC 6, TAC 12, and TAC 18 Gemini cationic surfactants in 15% DMSO-water were measured by plotting molar conductance (^) against concentration as shown in Eq. (1):

1 $$ \wedge = \frac{{1000 \times K_{s} }}{C} $$

where; ^ is the molar conductance of surfactant solution, ${K}_{s}$ the specific conductance of surfactant solutions, and C is a concentration of surfactant solution at different addition to water solvent.

The degree of ionization was calculated from Eq. (2):

2 $$ \propto = \frac{{S_{2} }}{{S_{1} }} $$

where S_1_ is the slope of the pre-micelle region and S_2_ is the slope of the post-micelle region.

Counter ion binding was calculated from Eq. (3):

3 $$ \beta = \left( {1 - \alpha } \right) $$

Then Gibbs free energy of micellization for surfactants as in monomers form ${\Delta G}_{mic (monomer)}$ and in dimers form $\Delta G_{{mic \left( {dimer} \right)}}$ was measure from Eq. (4) and (5).

4 $$ \Delta G_{{mic \left( {monomer} \right)}} = \left( {2 - \alpha } \right)RT\ln \left[ {CMC} \right] $$

5 $$ \Delta G_{{mic \left( {dimer} \right)}} = \left( {3 - 2\alpha } \right)RT\ln \left[ {CMC} \right] $$

where R is the universal gas constant and T is the absolute temperature which equals 298.15 K.

Limiting molar conductance of different surfactants was measured from (Eq. 6) where molar conductance was plotted against the square power of concentration of a surfactant solution at the premicellar curve.

6 $$ \wedge = \wedge_{0} - B\sqrt C $$

By using the data observed from all surfactants under study; TAC 6, TAC 12, and TAC 18 then applying Shedlovsky extrapolation as in Eq. (7)

7 $$\frac{1}{{\wedge}S({\mathcalligra{z}})}=\frac{1}{{{\wedge}}_{0}}+\frac{{K}_{a}C{\wedge}S({\mathcalligra{z}}){\gamma }_{i}^{2}}{{{\wedge}}_{o}^{2}}$$

where K_a_ is the association constant, γ_i_ is the activity coefficient estimated from the Debye − Huckel limiting law as modified by Robinson and Stokes and shedlovsky function S(ʑ) can be calculated from Eq. (8).

8 $$S\left({\mathcalligra{z}}\right)={[\frac{z}{2}+ \sqrt{1+9({\frac{Z}{2})}^{2}}]}^{2}$$

The association constants for TAC 6, TAC 12, and TAC 18 Gemini cationic surfactants were calculated and then the standard Gibbs free energy change of association was calculated for all Gemini cationic surfactants at 298.15 K According to Eq. (9).

9 $$ \Delta G_{a} = - 2.303 RT\log K_{a} $$

The degree of ionization, α, the counter ion binding, β, and the standard free energy of micellization, association constant (K_a_), and the standard free energy change of association (ΔG_a_) and micellization (ΔG_mic_) for the surfactants understudy in 15% DMSO–water solvent at 298.15 K are presented in (Table 2).

**Table 2:** The degree of ionization, α, the counter ion binding, β, and the standard free energy of micellization, association constant (K_a_), and the standard free energy change of association (ΔG_a_) and micellization (ΔG_mic_) for the surfactants understudy in 15% DMSO–water solvent at 298.15 K.

Surfactant abbreviation	$\propto$	$\beta$	${\Delta G}_{mic} (monomer)$(kJ/mol)	${\Delta G}_{mic} (Dimer)$(kJ/mol)	${K}_{a}$	${\Delta G}_{a}$(kJ/mol)
TAC 6	0.6108	0.3892	−25.947	−33.22	7720.78	−23.31
TAC 12	0.5854	0.4146	−27.775	−35.92	7790.10	−23.33
TAC 18	0.5521	0.4479	−30.946	−40.52	22049.9	−26.03

Two different factors affect the formation of micelles of all surfactants under study in a 15% DMSO-water solvent including; electrostatic repulsion of positive head groups which resist the formation of micelles and hydration of hydrocarbon chain which is more effective in the formation of micelles. Water molecules tended to interact with hydrocarbon chains in the surfactant tails and spacers between two head groups of surfactants with van Waals forces^41^. As summarized in (Table 2), the degree of ionization of counter ions was indicated from the ratio between the slopes after and before CMC of all surfactants^42^. A decrease in the slope after CMC was indicated due to the formation of micelles as shown in (Fig. 3) and a decrease in free monomers in the solution^43^. While binding of counter ions indicated more information about the aggregation of surfactants in the micellization process^44^. The binding constant for counter ions in the stern layer tended to be increased with the increment of hydrocarbon chain length while the spacer was the same for all surfactants. The increase in negative Gibbs free energy of micellization for both monomers and dimers for all surfactants proved the spontaneity of micellization and association processes as hydrocarbon chain length increased with increasing in hydrophobic interaction^45,46^. The association constant was found to be gradually increasing where TAC 18 acquired the highest value. This confirms the effect of the increase in hydrocarbon chains on the association of surfactants^47–49^.

#### Molar volume

The density of TAC 6, TAC 12, and TAC 18 Gemini cationic surfactants in molar concentration in 15% DMSO-water was measured at 298.15 K. Molar volumes (V_φ_) of all synthesized Gemini cationic surfactants under study were then calculated from the following Eq. (10):

10 $$ V_{\varphi } = \frac{M}{\rho } - \frac{1000}{m} \left[ {\frac{1}{{\rho^{o} }} - \frac{1}{\rho }} \right] $$

where; M is the molecular weight of the surfactant; m is the molar concentration of the surfactant in solution; and ρ and ρ° are the densities of the surfactant solution and 15% DMSO-water solvent, respectively.

The packing density (P); the relation between the van der Waals volume (V_w_) and the total molar volume of large molecules was found to have the same value^50^. so, van der Waals volumes (V_w_) of the surfactants can be calculated by using Eq. (11).

11 $$ P = \frac{{V_{w} }}{{V_{\varphi } }} = 0.661 \pm 0.017 $$

While the electrostriction volume which indicates the amount that the pure water solvent compresses can be calculated from Eq. (12)

12 $$ V_{E} = V_{w} - V_{\varphi } $$

**Figure 9:**
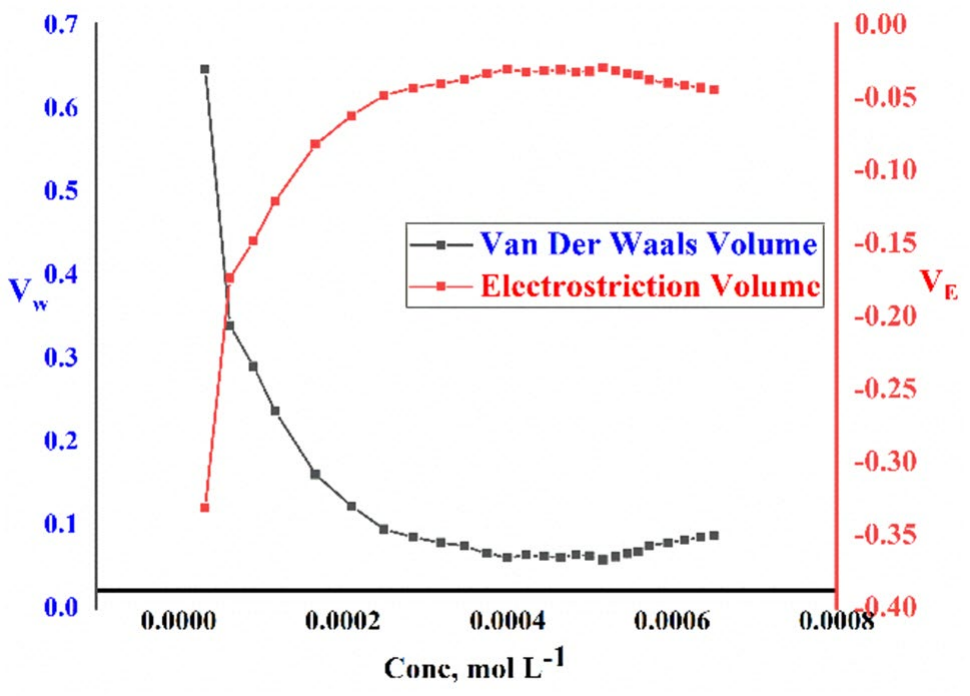
The relationship between molar concentration against Van der Waals Volume (V_w_) and electrostriction Volume (V_E_) for TAC 6 surfactants.

**Figure 10:**
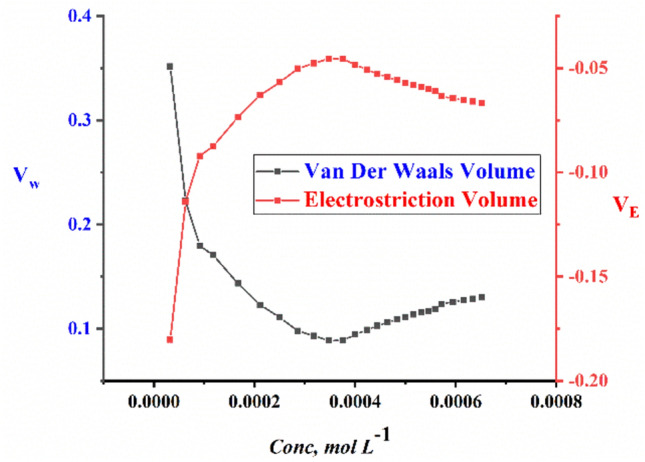
The relationship between molar concentration against Van der Waals Volume (V_w_) and electrostriction Volume (V_E_) for TAC 12 surfactants.

**Figure 11:**
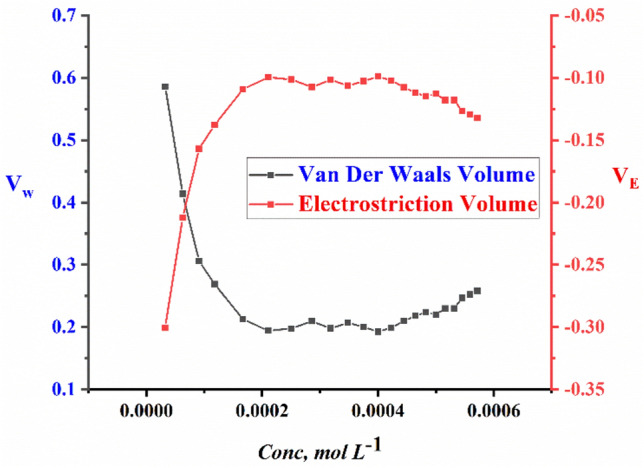
The relationship between molar concentration against Van der Waals Volume (V_w_) and electrostriction Volume (V_E_) for TAC 18 surfactants.

All calculated data including; electrostriction volume (${V}_{E}$) and Van der Waals volume ${V}_{w}$ of TAC 6, TAC 12, and TAC 18 Gemini cationic surfactants in 15% DMSO-water solvent at 298.15 K are shown in Figs. 9–11.

Through explaining the hydrophobic hydration of hydrocarbon chains of all surfactants with surrounding bounded water in the solvent. Which indicated a decrease in interaction between surfactants-solvent^51–53^. The values of van der Waals volume and electrostriction volume were calculated and indicated to decrease with the increment of concentration of all surfactants. Till reaching CMC, where dehydration process occurred and water molecules turned away from surfactant molecules^54^.

#### Modeling studying for density

**Figure 12:**
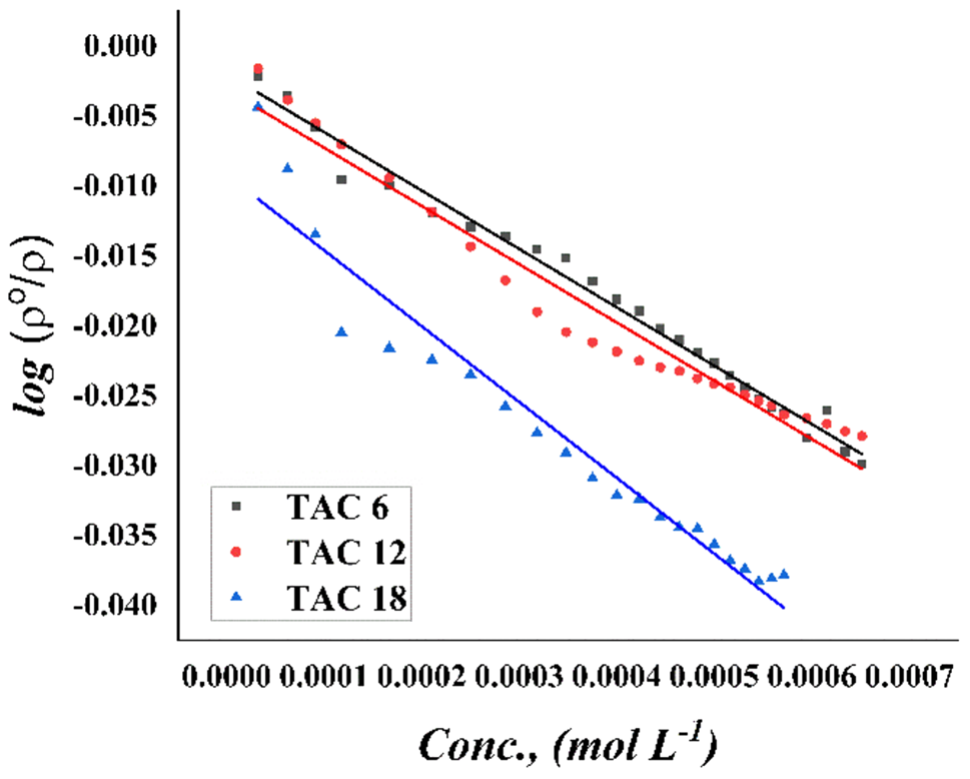
Modeling the effect of densities change with increasing concentration of surfactants TAC 6, TAC 12, and TAC 18.

The densities of all newly synthesized Gemini cationic surfactants under study; TAC 6, TAC 12, and TAC 18 were calculated at 15% DMSO-water solvent at 298.15 K. From measuring densities of 15% DMSO-water solvent and densities of different additions for all surfactants to 15% DMSO-water solvent, the ratio between densities of 15% DMSO-water and density of solution for different concentrations of all surfactants in the same solvent plotted against different concentrations of all newly surfactants at 298.15 K in molar concentration according to extended Setschenow Eq. (13)

13 $$ \log \frac{{\rho^{o} }}{\rho } = KC + K_{o} $$

where; ${\rho }^{o}$ is the density of all new surfactants in 15% DMSO-water for all synthesized surfactants TAC 6, TAC 12, and TAC 18. (K) is the Setschenow constant which is a measurable parameter for the effect of the concentration of all surfactants on the densities of surfactant solutions as shown in(Fig. 12).

It was found that the Setschenow constant increased but to a negative value which indicates an increase in the density of solution with the addition of different concentrations of different surfactants more rapidly compared with 15% DMSO-water solvent as shown in (Table 3). An increase in Setschenow constant may related to an increase in the molecular weight of all synthesized surfactants under study in the following order: TAC 18 > TAC 12 > TAC 6.

**Table 3:** The Setschenow parameter.

Surfactant abbreviation	K	K_o_	R^2^
TAC 6	−41.68	−0.00198	0.99
TAC 12	−45.78	−0.00311	0.96
TAC 18	−54.26	−0.00919	0.94

#### Refractive index

For all Gemini cationic surfactants under study; TAC 6, TAC 12, and TAC 18, the refractive index was indicated at a concentration equal to 1 × 10^−4^ mol L^−1^ in 15% DMSO-water solvent at 298.15 K.

Where; molar refraction (*R*_*m*_) for all surfactants was indicated by using their molar volumes and refractive indices from Eq. (14)

14 $$ R_{m} = \frac{{V_{\varphi } \left( {n^{2} - 1} \right)}}{{n^{2} + 2}} $$

While atomic polarization ($${P}_{A})$$ can be computed from Eq. (15)

15 $$ P_{A} = 1.05n^{2} $$

The polarizability of all surfactants understudies in 15% DMSO-water solvent at 298.15 K was calculated from Eq. (16)

16 $$ \alpha_{D} = \frac{{3V_{\varphi } \left( {n^{2} - 1} \right)}}{{4N\pi (n^{2} + 2)}} $$

where; where (N) is Avogadro’s number and (${\alpha }_{D}$) is the surfactant's polarizability.

Data summarized in (Table 4) indicate an increase in molar refraction, atomic polarizability, and polarizability at specific concentrations. This may be related to an increase in the hydrophobic solvation for larger surfactants. Where the stronger interaction between surfactants and solvent is indicated in the following arrangement: TAC 18 < TAC 12 < TAC 6^55^.

**Table 4:** refractive index (n_D_), molar refraction (${R}_{m}$), atomic polarizability (P_A_), and the polarizability (${\alpha }_{D}$) of TAC 6, TAC 12, and TAC 18 in 15% DMSO-water at 298.15 K.

Molar conc. mol L^−1^	Surfactants	n_D_	${R}_{m}$	${P}_{A}$(m^3^ mol^−1^)	${\alpha }_{D}$ E26
$1\times {10}^{-4}$	TAC 6	1.3606	0.0969	1.944	3.84
	TAC 12	1.3569	0.0795	1.933	3.82
	TAC 18	1.3518	0.1007	1.919	3.99

### Enhancing aggregation properties under salts effect

#### Critical micelle concentration detection by conductometric techniques

**Figure 13:**
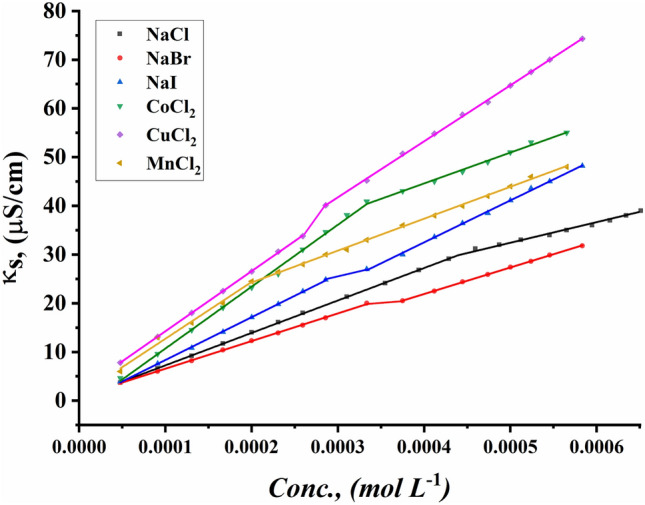
Conductivity vs. concentration for TAC 6 in 0.001 mol L^−1^ solution of different salts at 298.15 K.

**Figure 14:**
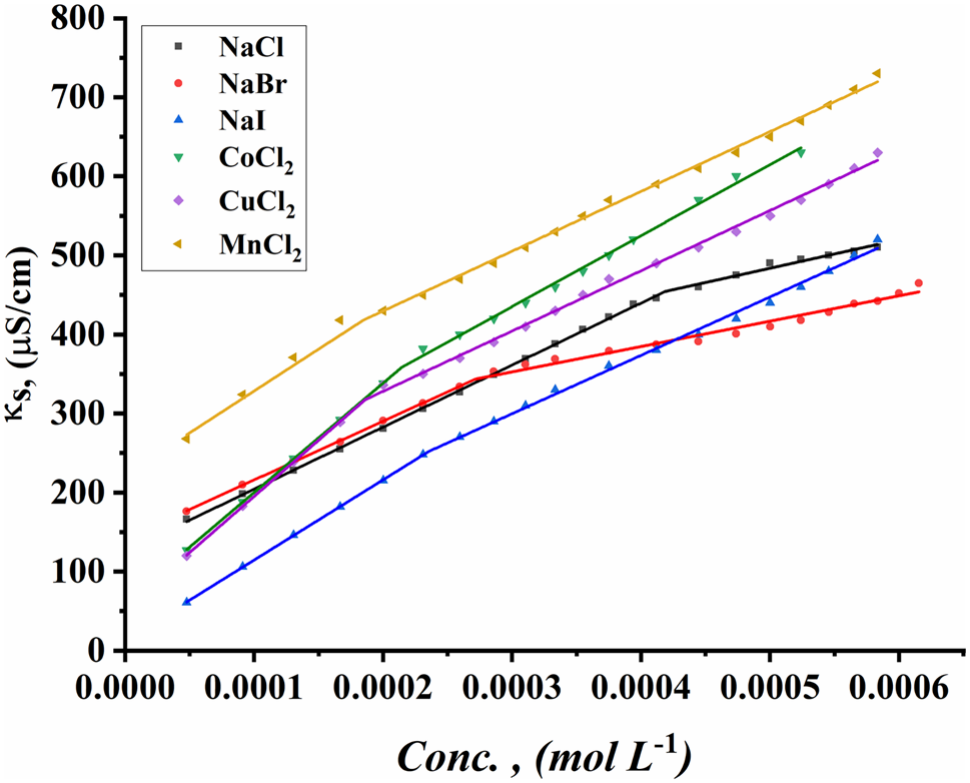
Conductivity vs. concentration for TAC 6 in 0.01 mol L^−1^ solution of different salts at 298.15 K.

**Figure 15:**
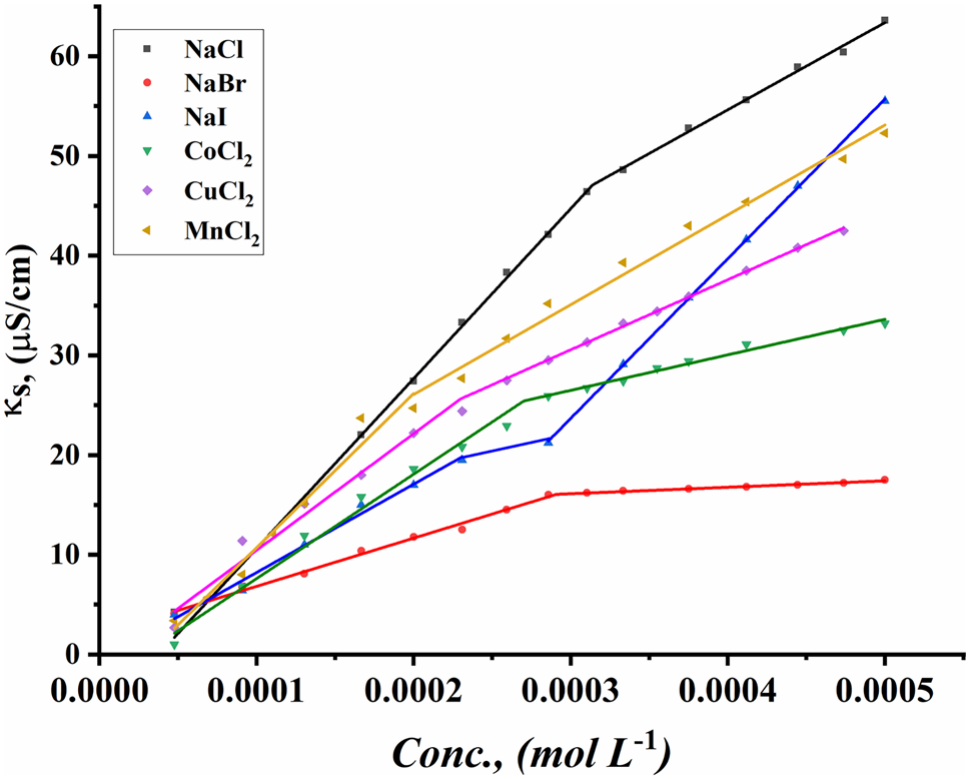
Conductivity vs. concentration for TAC 12 in 0.001 mol L^−1^ solution of different salts at 298.15 K.

**Figure 16:**
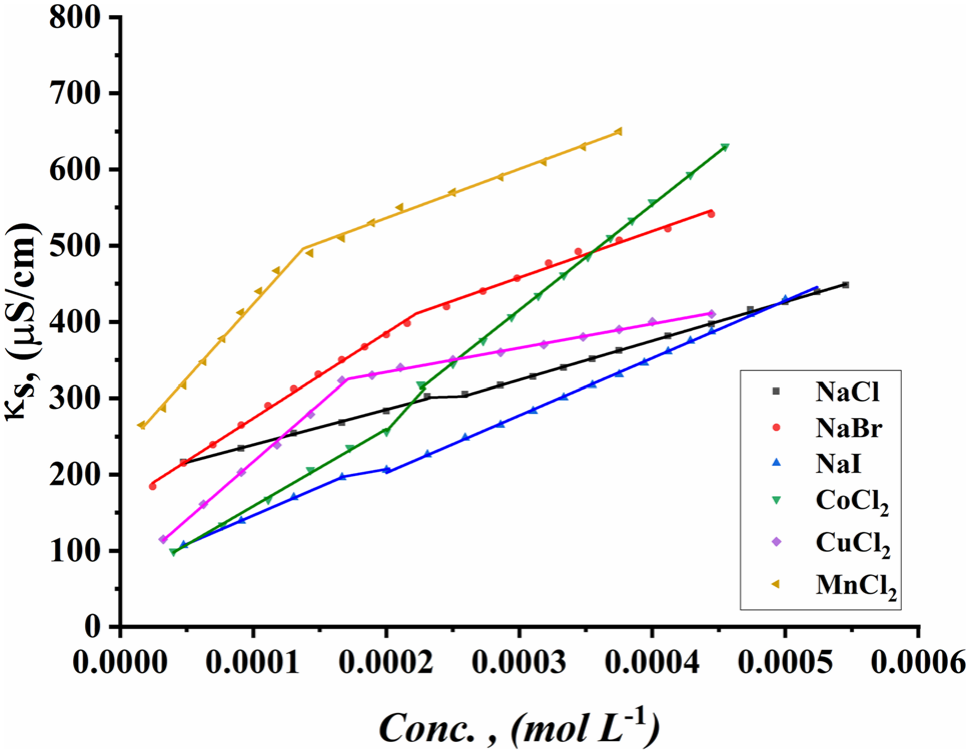
Conductivity vs. concentration for TAC 12 in 0.01 mol L^−1^ solution of different salts at 298.15 K.

**Figure 17:**
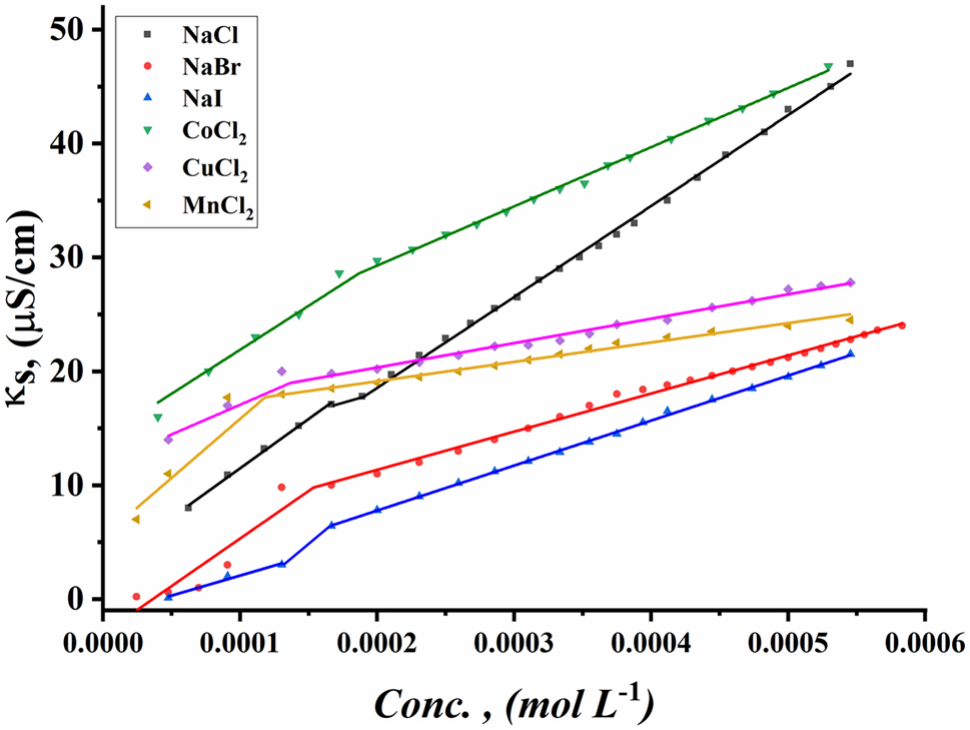
Conductivity vs. concentration for TAC 18 in 0.001 mol L^−1^ solution of different salts at 298.15 K.

**Figure 18:**
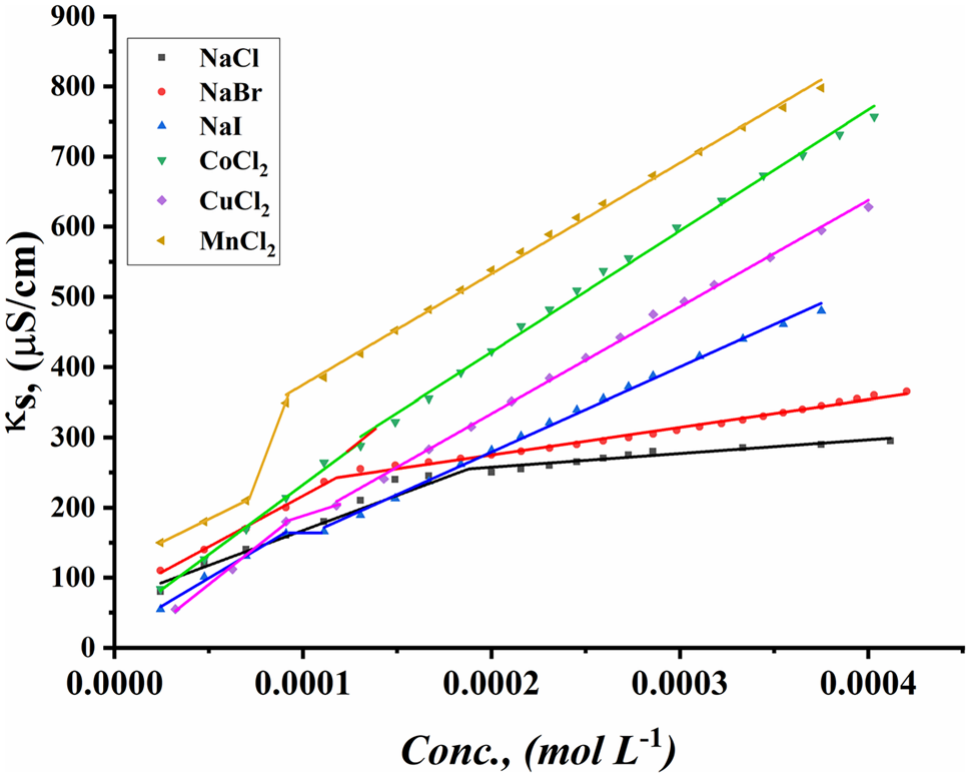
Conductivity vs. concentration for TAC 18 in 0.01 mol L^−1^ solution of different salts at 298.15 K.

The CMC values of all surfactants under study were indicated for the ternary system including all synthesized Gemini cationic surfactants under study, different sex inorganic salts, and 15% DMSO-water solvent at 298.15 K. Figures 13–18 indicate the relationship between the concentration of all surfactant solutions with different concentrations of six inorganic salts (0.001 and 0.01 mol L^−1^) in 15% DMSO-water solvent against solution conductivity. The selection of salt concentrations that were made in the study was based on choosing a single low concentration and a single high concentration of various inorganic salts to demonstrate the extent of their effect on the properties of the aggregation of surfactants under study^56–58^. Critical micelles concentration of all surfactants under study under the effect of salts are summarized in (Table 5).

**Table 5:** CMC of all surfactants TAC 6, TAC 12, and TAC 18 with different salt concentrations (0.001 M and 0.01 mol L^-1^) in 15% DMSO-water solvent at 298.15 K with conductometric technique.

Salt Conc. mol L^−1^	CMC (mol L^−1^)						
	Sur. abbr	NaCl	NaBr	NaI	CoCl_2_	CuCl_2_	MnCl_2_
0.001	TAC 6	0.000440	0.000354	0.000311	0.000333	0.000273	0.000198
	TAC 12	0.000312	0.000286	0.000257	0.000271	0.000229	0.000197
	TAC 18	0.000178	0.000155	0.000149	0.000189	0.000138	0.000121
0.01	TAC 6	0.000414	0.000287	0.000233	0.000213	0.000188	0.000186
	TAC 12	0.000245	0.000218	0.000187	0.000211	0.000168	0.000138
	TAC 18	0.000186	0.000116	0.000100	0.000135	0.000104	0.000081

The presence of different concentrations of six inorganic salts in 15% DMSO-water solvent simulated the salting out of all synthesized surfactants TAC 6, TAC 12, and TAC 18 in the solvent. This effect works as a catalyst for micelle formation through interaction between hydrocarbon chains attached to all surfactants^59^.

Where the presence of different six inorganic salts in 15% DMSO-water solvent increases the ionic strength of the surfactant solutions. While the CMC values of all surfactants highly decrease due to a decrease in electrostatic repulsion between intermolecular head groups^60^. Through studying the effect of counter ions effect on the aggregation of surfactants in the solvent under study, CMC values of all surfactants decreased with an increment of concentration of all six inorganic salts used as summarized in (Table 5).

While simulating the effect of counter ions on increasing the micelles formation of all surfactants, the CMC values of TAC 6, TAC 12, and TAC 18 indicated to decrease with the increasing in concentration of counter ion from different halide salts including NaCl, NaBr, NaI, MnCl_2_, CoCl_2,_ and CuCl_2_ at a given temperature 298.15 K^61^ Where the counter ions in these salts influence the balance between positive had groups of the surfactants and their counter ions through the micelles surface. This effect leads to free water bound to head groups of surfactants.

From the indication of the influence of the addition of six different inorganic salts to the surfactant solution, the decrement in the CMC of all surfactants used in this study can be summarized as the following 0.01 mol L^−1^ of all six salts < 0.001 mol L^−1^ of the same salts. This indicates the effect of increased concentration of salts on the formation of more stable micelles at low concentrations.

The trends of CMC for all surfactants indicated to be as the following CMC of NaCl < CMC of NaBr < CMC of CoCl_2_ < CMC of NaI < CMC of CuCl_2_ < CMC of MnCl_2_^56^. This arrangement may be related to the effect of the radius of each salt on its properties. Where the radius of salts affects the internuclear separation, ionic strength, ionization potential, lattice energy, and solubility of salts in different mediums. The radii in the Angstrom unit for six inorganic salts used in our study were indicated to be as the as the following NaCl = 1.81, NaBr = 1.96, NaI = 2.2, MnCl_2_ = 0.83, CoCl_2_ = 0.75 and CuCl_2_ = 0.73^62–64^.

By simulating the effect of cations of chloride common ion salts on the aggregation of all Gemini cationic surfactants under study, cobalt divalent cations were indicated to have the least effect on reducing CMC for all surfactants. This may be related to the decrease in radius of cobalt divalent ions which make ions much hydrated with solvent molecules through their higher affinity. Increasing the hydration of Co^2+^ ions leads to a decrease in the solvent molecules required for the hydrophobic hydration of surfactants^65^.

#### Detection of CMC under salt effect by refractive index measurement

**Figure 19:**
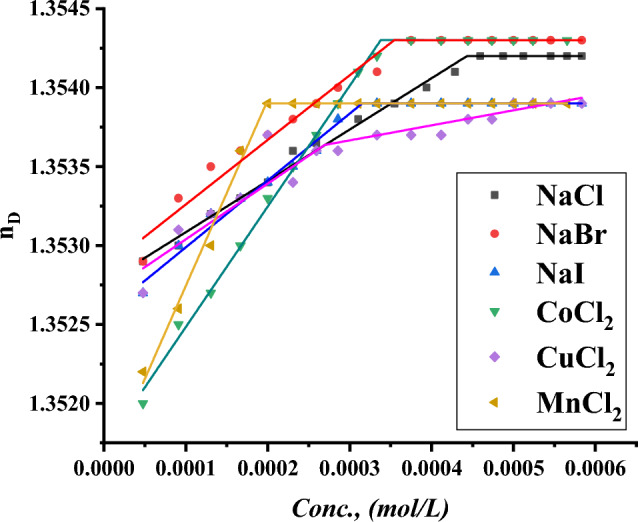
Refractive index vs. concentration for (TAC 6) in 0.001 mol L^−1^ solution of different salts at 298.15 K.

**Figure 20:**
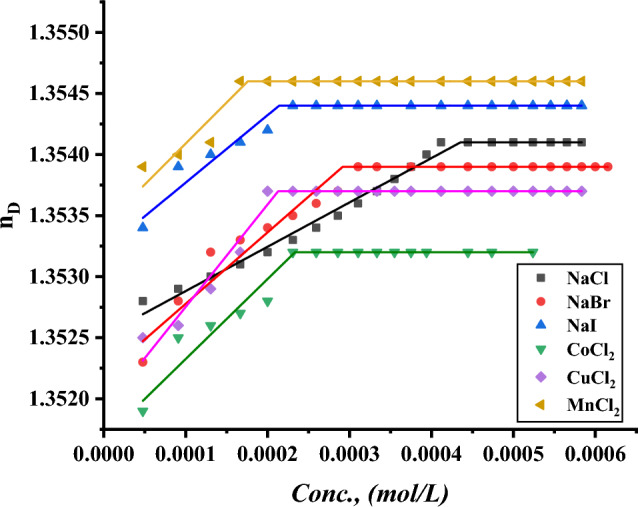
Refractive index vs. concentration for (TAC 6) in 0.01 mol L^−1^ solution of different salts at 298.15 K.

**Figure 21:**
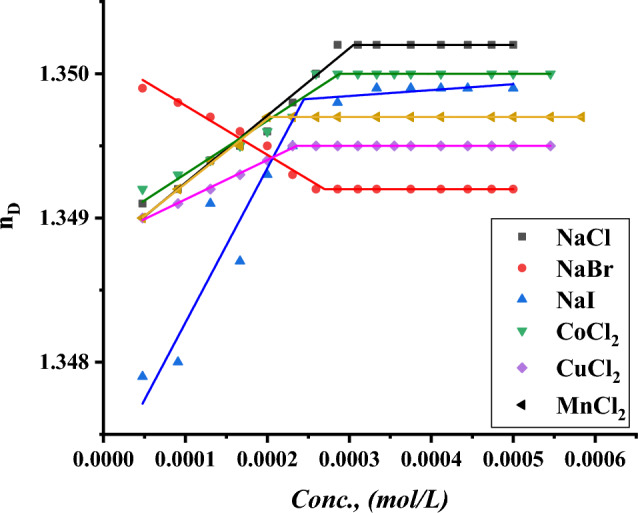
Refractive index vs. concentration for (TAC 12) in 0.001 mol L^−1^ solution of different salts at 298.15 K.

**Figure 22:**
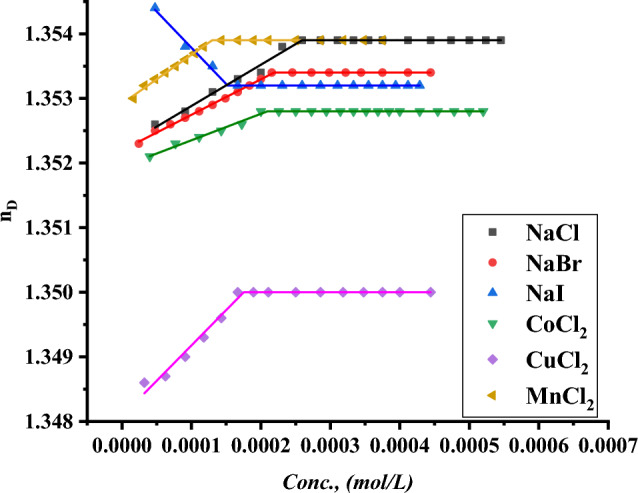
Refractive index vs. concentration for (TAC 12) in 0.01 mol L^−1^ solution of different salts at 298.15 K.

**Figure 23:**
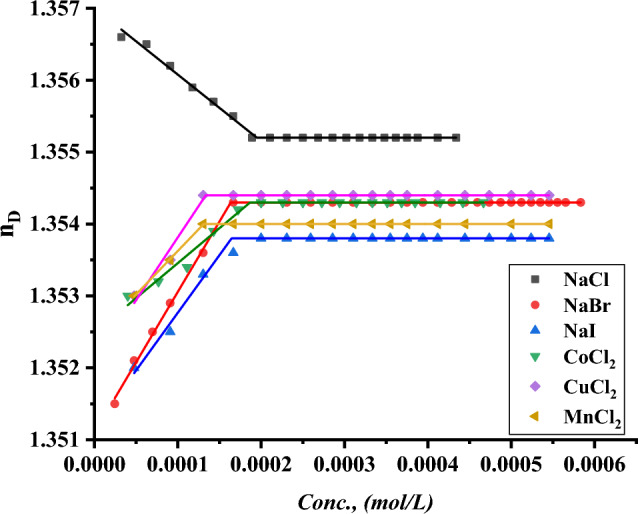
Refractive index vs. concentration for (TAC 18) in 0.001 mol L^−1^ solution of different salts at 298.15 K.

**Figure 24:**
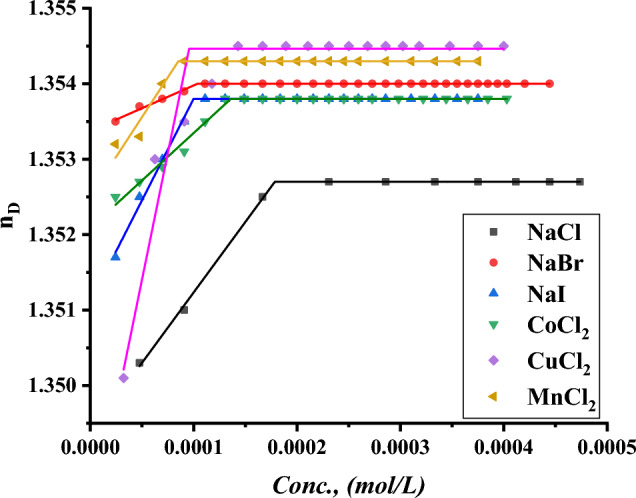
Refractive index vs. concentration for (TAC 18) in 0.01 mol L^-1^ solution of different salts at 298.15 K.

The CMC of all surfactants under study was indicated with the addition of a specific volume of all surfactants to a specific volume of two different concentrations of six inorganic salts. CMC of all indications was done by using a refractometer to detect the refractive index per each addition of surfactant to different mediums including all six inorganic salts where the refractive index of all surfactants in 0.001 and 0.01 mol L^−1^ of $NaI, NaBr, NaCl{, MnCl}_{2}, {CuCl}_{2} and {CoCl}_{2}$ plotted against the concentration of surfactant solution as shown in Figs. 19–24.

Refractive index measurement curves, where the refractive index for all surfactants is plotted against the concentration of solution with the addition of all surfactants in solution under different concentrations of inorganic salts.

As mentioned in Table 6, all six inorganic salts appeared to have the same effect on reducing the critical micelle concentration of all Gemini cationic surfactants. This may be related to the condensation of counter ions from different inorganic salts on the head groups of the Gemini cationic surfactant area in the micelles^66^.

**Table 6:** CMC of all surfactants in different salt solutions with 0.001 mol L^-1^ and 0.01 mol L^-1^ solution with 15% DMSO-water solvent at 298.15 K with refractive index techniques.

Conc. Mol L^−1^	CMC (Mol L^−1^)						
	Sur. abbr	NaCl	NaBr	NaI	CoCl_2_	CuCl_2_	MnCl_2_
0.001	TAC 6	0.000444	0.000355	0.000315	0.000337	0.000271	0.000200
	TAC 12	0.000306	0.000275	0.000246	0.000286	0.000232	0.000202
	TAC 18	0.000193	0.000163	0.000162	0.000187	0.000132	0.000129
0.01	TAC 6	0.000435	0.000289	0.000219	0.000231	0.000210	0.000174
	TAC 12	0.000257	0.000215	0.000152	0.000207	0.000171	0.000131
	TAC 18	0.000179	0.000107	0.000099	0.000136	0.000097	0.000087

The refractive indices of all surfactant solutions indicated an increase with the addition of different salts which are arranged as follows CMC of NaCl < CMC of NaBr < CMC of CoCl_2_ < CMC of NaI < CMC of CuCl_2_ < CMC of MnCl_2_. The increment in the radius of counter ions condensate on the micelle surface leads to an increase in the size of micelles formed. Where iodide counter ions acquired the largest effect on reducing CMC of all used surfactants. Where the radius of iodide < bromide < chloride^26^.

While increase in the salt concentration leads to an increase in the condensation of more counter ions on micelle surfaces. This effect is responsible for the decreasing of CMC of all surfactants at 0.01 mol L^−1^ compared to CMC at 0.001 mol L^−1^ for the same surfactants^27^.

#### Thermodynamic parameters from conductivity measurements under the effect of salts

Indications of the degree of ionization, and counter ion binding for all Gemini cationic surfactants TAC 6, TAC 12, and TAC 18 were calculated using the same Eqs. (2)–(3) mentioned before. Then micellization Gibbs free energy was also calculated and compared between all different inorganic salts at two different concentrations 0.001 and 0.01 mol L^−1^ according to Eq. (4) at 298.15 K. Using all previous calculations also the association constant and Gibbs free energy of association were indicated in Eqs. (7)–(9). All calculations related to conductometric measurement are summarized in(Table 7).

**Table 7:** the degree of ionization, (α) the counter ion binding, (β) and the standard free energy of micellization, the limiting molar conductance (Λ_°_), association constant (K_a_), and the standard free energy change of association (ΔG_a_) and micellization (ΔG_mic._) for the surfactants understudy in 0.001 M and 0.01 M solutions of different inorganic salts 15% DMSO –water solvent at 298.15 K.

Sur. abbr	Salts Conc. mol L^−1^	Salt name	$\propto$	$\beta$	${\Delta G}_{mic}$(kJ/mol)	${K}_{a}$	${\Delta G}_{a}$(kJ/mol)
TAC 6	0.001	NaCl	0.6351	0.3649	−26.149	954688	−34.14
		NaBr	0.7774	0.2226	−24.081	1288134	−34.88
		NaI	0.8799	0.1201	−22.423	1021480	−34.31
		CoCl_2_	0.6574	0.3426	−26.648	1001975	−34.26
		CuCl_2_	0.7278	0.2722	−25.878	1260804	−34.83
		MnCl_2_	0.8615	0.1385	−24.064	1304514	−34.91
	0.01	NaCl	0.7452	0.2548	−24.230	1224.082	−35.64
		NaBr	0.7953	0.2047	−24.357	1899.266	−35.69
		NaI	0.8074	0.1926	−24.728	1085.882	−37.75
		CoCl_2_	0.6494	0.3506	−28.305	1367.773	−38.61
		CuCl_2_	0.7474	0.2526	−26.637	1955.179	−37.44
		MnCl_2_	0.8802	0.1198	−23.843	3600.652	−35.60
TAC 12	0.001	NaCl	0.6234	0.3766	−27.546	1155386	−34.61
		NaBr	0.7744	0.2256	−24.788	1166055	−34.63
		NaI	0.6014	0.3986	−28.659	2932613	−36.92
		CoCl_2_	0.5902	0.4098	−28.704	1290646	−34.88
		CuCl_2_	0.6512	0.3488	−28.024	1377800	−35.05
		MnCl_2_	0.7227	0.2773	−27.015	2423315	−36.45
	0.01	NaCl	0.9019	0.0981	−22.630	3424217	−37.30
		NaBr	0.8592	0.1408	−23.841	2784844	−35.69
		NaI	0.9868	0.0132	−21.560	3179583	−37.12
		CoCl_2_	0.7470	0.2530	−26.287	2066081	−36.05
		CuCl_2_	0.7305	0.2695	−27.352	4140233	−37.77
		MnCl_2_	0.7635	0.2365	−27.243	2764713	−36.77
TAC 18	0.001	NaCl	0.9221	0.0779	−23.068	1402412	−35.09
		NaBr	0.6261	0.3739	−29.875	2528953	−36.55
		NaI	0.8899	0.1101	−24.247	2778431	−36.79
		CoCl_2_	0.6738	0.3262	−28.185	2827519	−36.83
		CuCl_2_	0.6675	0.3325	−29.358	2015745	−35.99
		MnCl_2_	0.7181	0.2819	−28.660	2587122	−35.99
	0.01	NaCl	0.9908	0.0092	−21.488	3245239	−37.17
		NaBr	0.8679	0.1321	−25.429	2154839	−36.16
		NaI	0.9313	0.0687	−24.399	3219911	−37.15
		CoCl_2_	0.8671	0.1329	−25.022	5524692	−38.49
		CuCl_2_	0.7142	0.2858	−29.231	7923313	−39.38
		MnCl_2_	0.8357	0.1643	−27.191	3118896	−37.07

As observed in Table 7 all degrees of ionization of all surfactants in different mediums include two concentrations of six inorganic salts (0.001 and 0.01 mol L^−1^) observed to increase, respectively, with an increase in the addition of all salts. This effect may be related to an increment in solution ionic strength with an increase in the number of unbounded counter ions^67^. Increasing salt concentration for all salts directly proportional with ionic strength and therefore increases the degree of ionization of the surfactant solution in the following arrangement 0.01 mol L^−1^ of different salts < 0.001 mol L^−1^ of the same salts.

There was a reduction in the magnitude of Gibbs free energy of both micellization and association of all surfactants with the addition of all six inorganic salts as shown in (Table 7). This indication proved a decrease in the amount of energy required to transfer monomers of all surfactants TAC 6, TAC 12, and TAC 18 from bulk solution to micelles.

Negative values of Gibbs free energy of micellization and association proved an increment in the spontaneous nature of these two processes in the presence of all six inorganic salts. This may be related to the fact that the presence of all six inorganic salts improved the micelles formed in all surfactant solutions and made them more stable^68^.

**Figure 25:**
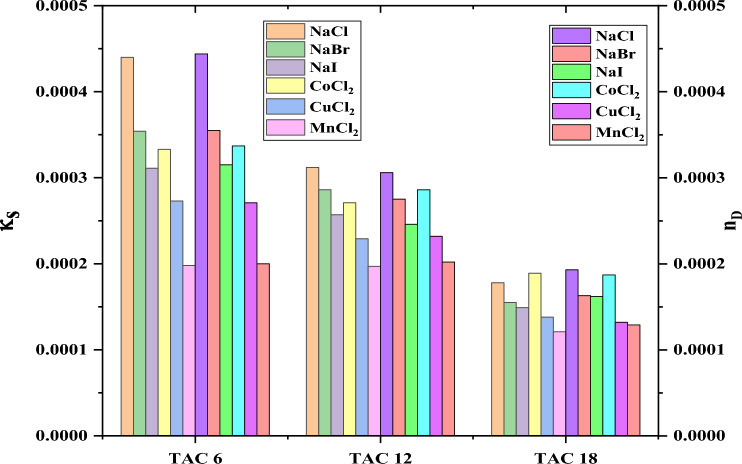
Comparison between CMC of different surfactants TAC 6, TAC 12, and TAC 18 under the effect of different salts at 0.001 mol L^−1^ using conductivity and refractive index measurement.

**Figure 26:**
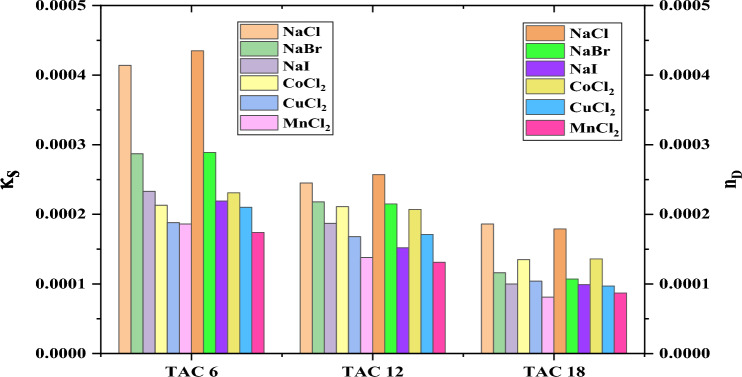
Comparison between CMC of different surfactants TAC 6, TAC 12, and TAC 18 under the effect of different salts at 0.01 mol L^−1^ using conductivity and refractive index measurement.

The comparison between the effect of two concentrations of different inorganic salts on all surfactants under study indicated by conductivity and refractive index measurements has been referred to in Figs. 25, 26.

## Conclusion

This study focused on investigating the micellization behavior of three newly synthesized Gemini cationic surfactants (TAC 6, TAC 12, and TAC 18) in a 15% DMSO-water solvent system at 298.15 K. Some techniques including conductivity, refractive index, density, molar volume, and surface tension measurements were employed to elucidate the thermodynamic parameters during the aggregation process, both in the absence and presence of various concentrations of inorganic salts (NaCl, NaBr, NaI, CoCl_2_, CuCl_2_, and MnCl_2_).

The critical micelle concentration (CMC) values obtained from all techniques showed good agreement and revealed a trend where micellization increased with the hydrocarbon chain length of the surfactants: CMC _(TAC 18)_ < CMC _(TAC 12)_ < CMC _(TAC 6)_ in the 15% DMSO-water solvent at 298.15 K.

The influence of salt addition on the reduction CMC of the surfactants followed the order: NaCl < NaBr < NaI < CoCl_2_ < CuCl_2_ < MnCl_2_. Increasing salt concentration led to enhanced micellization and lower CMC values for all surfactants, with CMC (0.01 mol L^−1^) being lower than CMC (0.001 mol L^−1^). Furthermore, the more negative values of Gibbs free energies of association (ΔG_ass._) and micellization (ΔG_mic_) in the presence of different salts indicated the spontaneous formation of more stable micelles. The degree of ionization (α) of all surfactants also increased in the presence of salts, confirming the easier formation of micelles at lower concentrations.

Overall, these findings underscore the significant role of solvent composition and salt presence in modulating the aggregation behavior of Gemini cationic surfactants. Such insights are crucial for optimizing surfactant applications in various industrial and technological fields, including pharmaceuticals, cosmetics, and chemical processes.

## Data Availability

Raw data were generated at Faculty of Science, Port-Said University, Egypt. Derived data supporting the findings of this study are available from the corresponding author, Prof. Dr. Farid I. El-Dossoki, on request.
